# Approved Remedies in the Treatment of Acute Epidemic Bronchitis (Influenza)

**Published:** 1890-03

**Authors:** 


### APPROVED REMEDIES IN THE TREATMENT OF ACUTE EPIDEMIC BRONCHITIS (INFLUENZA.)

Accumulated experience with the epidemic, familiarly known as "la grippe," has already afforded ample opportunity to correctly guage the efficiency of various modes of treatment suggested, and establish the value of different remedies employed to correct the abnormal symptoms. Of those which have proven themselves most useful, Parke, Davis & Co. call attention to some combinations which are especially adapted to meet the prominent indications.

The catarrhal affections of the respiratory mucous membrane resolve themselves into several distinct phases: I. Bronchital catarrh. This is alleviated by the employment of the following formula, for which we have selected the name "Syrup White Pine, Compound." Each fluidounce represents:

| | | |
|---|---|---|
| White pine bark - - - | 30 | grains. |
| Wild cherry bark - - - | 30 | grains. |
| Sassafras bark - - - | 2 | grains. |
| Bloodroot - - - - | 3 1-2 | gaains. |
| Balm-of-Gilead buds - - | 4 | grains. |
| Chloroform - - - - | 4 | minims. |
| Spikenard - - - - | 4 | grains. |
| Morphine acetate - - - | 3-16 | grains. |

One of the most marked indications for treatment is to restore the dry mucous membrane to a condition of normal secretion, to overcome the interference with respiration by stimulating the respiratory centers, and to allay the inflammation and irritation by sedatives and demulcents.

The formula which we offer under the name of bronchial sedative, has also been found admirably adapted for meeting the threefold indications specified. It has been widely used in the hospitals of this country and Europe, as well as in private practice. It is palatable and readily taken by children, and more faithfully by adults than the nauseous expectorant mixtures, so often given. Unlike the latter, it does not interfere with digestion, and may be administered as required without developing any outward symptoms. The physician will, of course, modify the formula and dose, when necessary, to meet the requirements of the individual case.

Each fluid ounce contains:

| | |
|---|---|
| Ammonia chloride- - - | 30 grains. |
| Fluid tolu, soluble - - | 8 minims. |
| Fluid opium, camphorated - | 4 minims. |
| Elixir licorice, aromatic, q. s., ad | 1 fluidounce. |

